# Mussel-Inspired Calcium Alginate/Polyacrylamide Dual Network Hydrogel: A Physical Barrier to Prevent Postoperative Re-Adhesion

**DOI:** 10.3390/polym15234498

**Published:** 2023-11-23

**Authors:** Zekun Su, Beibei Xue, Chang Xu, Xufeng Dong

**Affiliations:** 1 School of Materials Science and Engineering, Dalian University of Technology, Dalian 116024, China; suzekun@mail.dlut.edu.cn (Z.S.); xbb@mail.dlut.edu.cn (B.X.); 2 Institute of Cardio-Cerebrovascular Medicine, Central Hospital of Dalian University of Technology, Dalian 116089, China

**Keywords:** anti-adhesion, dual-network hydrogel, polydopamine, elasticity modulus, tissue adhesion

## Abstract

Intrauterine adhesions (IUA) has become one of the main causes of female infertility. How to effectively prevent postoperative re-adhesion has become a clinical challenge. In this study, a mussel-inspired dual-network hydrogel was proposed for the postoperative anti-adhesion of IUA. First, a calcium alginate/polyacrylamide (CA-PAM) hydrogel was prepared via covalent and Ca^2+^ cross-linking. Benefiting from abundant phenolic hydroxyl groups, polydopamine (PDA) was introduced to further enhance the adhesion ability and biocompatibility. This CA-PAM hydrogel immersed in 10 mg/mL dopamine solution possessed remarkable mechanical strength (elastic modulus > 5 kPa) and super stretchability (with a breaking elongation of 720%). At the same time, it showed excellent adhesion (more than 6 kPa). Surprisingly, the coagulation index of the hydrogel was 27.27 ± 4.91, demonstrating attractive coagulation performance in vitro and the potential for rapid hemostasis after surgery.

## 1. Introduction

Intrauterine adhesion (IUA) is a common gynecological disease caused by endometrial injury and the abnormal proliferation of fibrous tissue, which has become one of the main causes of female infertility [1,2,3]. At present, the main treatment for IUA is the transcervical resection of adhesions (TCRA), which uses miniature scissors to separate the adhesion [4]. However, since TCRA is still a form hysteroscopic surgery in nature, mechanical damage will be caused again during the separation process; beyond that, the incidence of postoperative re-adhesion can reach up to 62.5% [1]. Therefore, how to prevent re-adhesion safely and effectively after TCRA has become a key issue in clinical treatment.

At present, physical barriers such as an intrauterine device and films are mainly used in clinical practice to prevent re-adhesion [5,6,7]. However, due to various reasons, such as poor biocompatibility and mismatch of mechanical properties, the treatment effect is limited, which can only alleviate or reduce adhesion to a certain extent [8,9,10,11]. Hydrogels are extremely hydrophilic three-dimensional networks of gel that can rapidly swell in water and retain a large amount of water without dissolving [12,13,14], and their physical and chemical properties can be adjusted so as to achieve a strong ability to match biological tissues [15,16]. Nowadays, some kinds of hydrogels have been used in clinical cases to prevent re-adhesion after TCRA surgery [17]. As a commonly used intraluminal anti-adhesion hydrogel, alginate has good biocompatibility and anti-bacterial properties, but its poor mechanical properties cannot achieve complete wound separation, which may lead to re-adhesion [18,19]. Because of this, further clinical application is limited [20]. To meet the demand for stronger mechanical properties, Suo et al. [21] designed a double-network hydrogel that combines two single-network gels—calcium ion cross-linked alginate and polyacrylamide—to break through the disadvantage of the poor mechanical properties of hydrogels. However, the hydrogel was not adhesive and struggled to remain in the same position for a certain time.

In order to stabilize the anti-adhesion hydrogel with excellent mechanical properties on the uterine cavity wound good tissue adhesion is also required. For this purpose, researchers have begun to focus on mussel biomimetic materials in recent years [22,23]. The strong adhesion ability of mussels is due to their unique adhesion proteins, in which a large number of catechol groups can allow mussels to adsorb to objects through hydrogen bonds [24]. PDA is obtained via the oxidation and self-polymerization of DA in a weak alkaline environment, and the structure of PDA contains a large number of catechol groups, which is relatively similar to that of the adhesion proteins and can achieve the purpose of strong adhesion [25]. At the same time, the surface modification of materials with dopamine can also bring strong biocompatibility, degradation, hemostasis, and other comprehensive properties [26,27]. It seems that the introduction of PDA into dual-network hydrogels can combine excellent mechanical properties with adhesion ability.

Based on this, this study aims to synthesize a PDA dopation-modified calcium alginate/polyacrylamide double-network hydrogel and optimize its mechanical properties and tissue adhesion ability by adjusting the immersion concentration and doping amount of PDA. At the same time, the intrauterine anti-adhesion hydrogel needs to possess certain swelling capacity, stable degradation rate, and good hemostatic ability and biocompatibility. These properties were tested separately, and the application prospect of the hydrogel as an anti-adhesion hydrogel in preventing re-adhesion after TCRA was systematically studied and evaluated. The potential of the hydrogel, using as an anti-adhesion hydrogel for clinical application, is expected.

## 2. Materials and Methods

### 2.1. Materials

Acrylamide (AM), sodium alginate (SA, M/G = 1:2), dopamine hydrochloride (DA), N,N′-bis(acrylyl)cystamine (BAC), ammonium persulfate (APS), N,N,N-,N-tetramethylenediamine (TEMED), and trimethylol aminomethane (Tris) were purchased from Shanghai Macklin Biochemical Co., Ltd. (Shanghai, China), and anhydrous calcium chloride(CaCl_2_) was purchased from Tianjin Kemiou Chemical Reagent Co. (Tianjin, China) Deionized (DI) water was home-made in the laboratory.

### 2.2. Preparation of PDA-CA-PAM Hydrogels

**Scheme 1:**
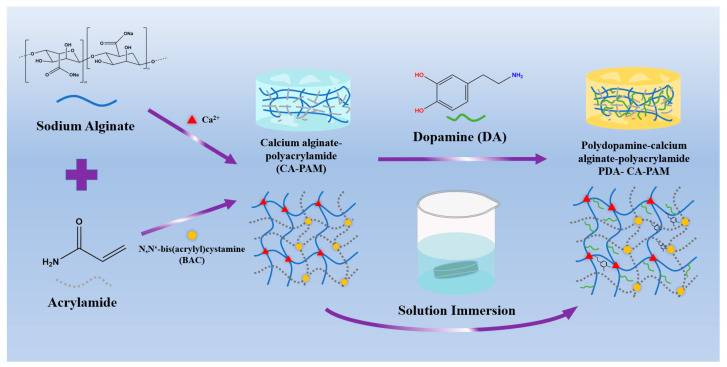
Schematic representation of the formation of an anti-adhesion hydrogel in preventing re-adhesion after TCRA.

As shown in Scheme 1, the hydrogels were prepared via the following two steps: first, AM (3.6 g), BAC (0.0132 g), and SA (0.6 g) were dissolved in 30 mL DI water under ice bath; then, APS and TEMED were added into the mixture and stirred for another 15 min. The hydrogel was left for 24 h and then immersed in 0.01 M CaCl_2_ solution for another 6 h to obtain CA-PAM hydrogel. Then, 5 mg/mL DA was dissolved in 0.01 M Tris solution with pH = 8.5 to form the dopamine solution. The CA-PAM hydrogel was then immersed into the solution and stirred for 24 h to obtain the 5 PDA-CA-PAM hydrogel. Using the same method, 10 and 20 mg/mL DA solutions were prepared to obtain 10 PDA-CA-PAM and 20 PDA-CA-PAM hydrogels.

### 2.3. Characterization

The chemical structure of the hydrogels was verified via Fourier transform infrared spectroscopy Nicolet iS20 (FTIR, Thermo Scientific, Waltham, MA, USA) in the range of 4000–500 cm^−1^. The morphologies were observed via field emission scanning electron microscope IT800-SHL (FESEM, JEOL, Tokyo, Japan) at a voltage of 10 kV. The mechanical properties were tested via a universal testing machine (HY-0580, Heng Wing Precision Instrument Co., Ltd., Shanghai, China) with the speed of 30 mm/min. The compression tests were carried out using the cylindrical hydrogels, which were compressed to 75% strain at a speed of 50 mm/min. Rheology analyses were performed using the MCR301 rheometer (Anton-Paar, Graz, Austria) at 37 °C with a smooth plate. The hydrogels were cut into circular disks with a diameter of 13 mm and a thickness of 2 mm using a cutting tool. Strain–sweep oscillation tests within the range of 0.01% to 100% were performed at 1 Hz frequency. The functions describing the changes in storage modulus G′ and loss modulus G″ with respect to strain were recorded.

### 2.4. Adhesion Measurements

The adhesive strength of the PDA-CA-PAM hydrogel was investigated via the lap shear method following a previously reported study with some modifications [28]. The fresh hydrogel was cut into 22 × 22 × 2 mm and adhered to the middle of two pieces of pig skin, which were attached to two identical slides. Then, the slide was stretched to failure at a speed of 5 mm/min via a HY-0580 electronic universal testing machine. The adhesive strength was calculated by dividing the maximum force over the area of the adhesive overlap. All samples were tested three times.

### 2.5. Swelling Behavior

Before the test, the initial weight (W_0_) was weighed, and then the PDA-CA-PAM hydrogel was soaked in PBS at 37 °C and removed at regular intervals. Before every weighing, filter paper will be used to remove the water on the surface, and the weight of the hydrogel after the swelling time (t), W_t_, will be obtained. The swelling ratio was calculated via Formula (1):

(1) $$\mathrm{Swelling}\;\mathrm{ratio}\;\left(\%\right)=\frac{W_{t}-W_{0}}{W_{0}}\;\times \;100\%.$$

### 2.6. Biodegradation Behavior

The initial weight W_0_ of the PDA-CA-PAM hydrogel was weighed, and then the hydrogel was soaked in PBS and 20 μM cysteine and placed in a constant-temperature shaker at 37 °C. The samples were periodically removed and washed with water then freeze-dried for 48 h and weighed again, recorded as W_d_. The biodegradation was measured as the percent mass loss of the hydrogel samples, calculated via Formula (2):

(2) $$\mathrm{Weight}\;\mathrm{loss}\;\left(\%\right)=\frac{W_{0}-W_{d}}{W_{0}}\;\times \;100\%.$$

### 2.7. Hydrophilicity Assay

The surface hydrophilicity of the hydrogel was tested using a contact angle meter (DSA100, KRUSS, Hamburg, Germany). A total of 2 μL DI water was dropped on the surface of the sample, the image after the droplet touched the surface was recorded, and the water contact angle was calculated.

### 2.8. Hemolysis Assay

Fresh goat blood was diluted with saline. The PDA-CA-PAM hydrogel was rinsed and immersed in centrifuge tube containing 10 mL saline. The centrifuge tube was incubated at 37 °C for 30 min, and then 0.2 mL diluted blood was added and incubated at 37 °C for 60 min. Saline was used as negative control group, while DI water was used as a positive control group. All tubes were centrifuged at 3000 rpm for 5 min, collecting the supernatant, and the optical density value (OD) was recorded at 540 nm microplate reader. The hemolysis rate was calculated via Formula (3):

(3) $$\mathrm{Hemolysis}\;\left(\%\right)=\frac{{\mathrm{OD}}_{\mathrm{Sample}\;}-{\;\mathrm{OD}}_{\mathrm{Negative}}}{{\mathrm{OD}}_{\mathrm{Positive}}-{\mathrm{OD}}_{\mathrm{Negative}}}\;\times \;100\%.$$

### 2.9. Hemostasis Assay

Goat blood was diluted with CaCl_2_ solution at a ratio of 1:9 and then dropped on the surface of the PDA-CA-PAM hydrogel. The dishes were transferred to an incubator for 5 min at 37 °C. After the reaction, DI water was added to dissolve the uncoagulated blood cells. The 100 μL supernatant was added to the 96-well plate, and the OD value at 540 nm was read with an enzyme marker. The blank dish served as the control group. The final coagulation index (BCI) value was calculated by quantifying the percentage of blood cells that had not coagulated into blood clots, referring to Formula (4):

(4) $$\mathrm{BCI}\;(\%)=\frac{{\mathrm{OD}}_{\mathrm{Sample}}}{{\mathrm{OD}}_{\mathrm{Control}}}\;\times \;100\%.$$

### 2.10. Cytocompatibility

NIH/3T3 cells were cultured in Dulbecco’s modified Eagle’s medium (DMEM) supplemented with 10% fetal bovine serum (FBS), 100 U/mL penicillin G, and 100 mg/mL streptomyces at 37 °C and 5% CO_2_. The 2 × 10^4^ cells/mL NIH/3T3 were inoculated into a 24-well plate containing PDA-CA-PAM hydrogel, cultured for 1 d, 2 d, and 3 d. For the corresponding points, the cells were fixed with 4% paraformaldehyde for 10 min, and then rinsed again 3 times. Finally, 0.1% TritonX-100 was used to permeate the cells. The cytoskeleton was stained with AcN-Trackergreen-488 and observed via fluorescence microscope (Olympus, Tokyo, Japan).

The proliferation of the NIH/3T3 cells in hydrogel was accessed via CCK-8 assay. In short, 1 × 10^4^ cells/mL of the cells were seeded and cultured overnight. The cell proliferation capacity was detected after 1, 2, and 3 days of incubation using cell counting kit-8 (CCK-8, Solarbio, Beijing, China). The control group consisted of cells and culture medium, while the blank group only contained culture medium. A total of 100 μL of fresh medium and 10 μL of CCK-8 solution were added to each well and incubated at 37 °C for 4 h. Thereafter, the OD value at 450 nm was measured, and the cell viability was calculated via Formula (5):

(5) $$\mathrm{Cell}\;\mathrm{viability}\;\left(\%\right)=\frac{{\mathrm{OD}}_{\mathrm{Sample}}\;-\;{\mathrm{OD}}_{\mathrm{Blank}}}{{\mathrm{OD}}_{\mathrm{Control}}\;-\;{\mathrm{OD}}_{\mathrm{Blank}}}\;\times \;100\%.$$

### 2.11. Statistical Analysis

All experiments were repeated three times, and data were analyzed using SPSS13.0 software. All data are given as the mean ± standard deviation (SD), and *p* < 0.05 shows that the results were statistically significant.

## 3. Results and Discussion

### 3.1. Preparation and Characterization of PDA-CA-PAM Hydrogel

The synthesis process of the hydrogel in this study is illustrated in Scheme 1. Based on the synthesis of the CA-PAM, the double-network hydrogel was immersed in dopamine (DA) solution. Polydopamine (PDA) chains were formed via self-polymerization in a weakly alkaline environment and then introduced into the gel network. Chemical cross-linking between molecular chains provided a rigid network, and the introduced PDA chain contained a significant number of hydroxyphenyl groups. For easy description, the PDA-CA-PAM hydrogel obtained via immersion in 5 mg/mL DA solution will be referred to as 5 PDA-CA-PAM for short. Similarly, 10 mg/mL and 20 mg/mL were named 10 PDA-CA-PAM and 20 PDA-CA-PAM, respectively.

**Figure 1:**
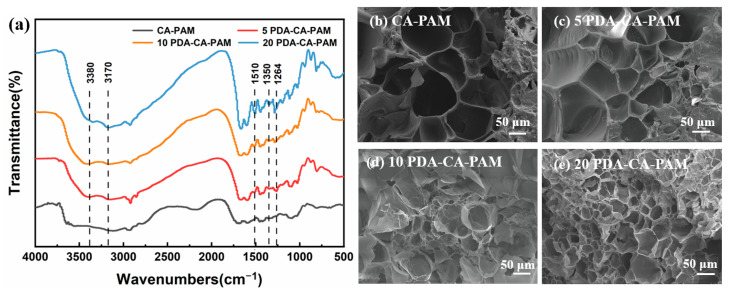
(**a**) FTIR spectra of CA-PAM hydrogel and PDA-CA-PAM hydrogels. SEM images of (**b**) CA-PAM hydrogel and PDA-CA-PAM hydrogel obtained via immersion with DA concentrations of (**c**) 5, (**d**) 10, and (**e**) 20 mg/mL.

To demonstrate that PDA was successfully doped, the chemical structure inside the hydrogel was characterized using FTIR. As shown in Figure 1a, compared with the CA-PAM hydrogel, the PDA-CA-PAM hydrogel showed a peak at 1264 cm^−1^, which corresponded to the stretching vibration of C-N in PDA aniline, and this band’s appearance implied the interaction between the NH_2_ group of PAM and the catechol groups of PDA. In addition, the characteristic peaks of the aromatic ring belonging to PDA also appeared at 1510 cm^−1^ and 1350 cm^−1^. The absorption peaks located at 3380 cm^−1^ and 3170 cm^−1^ were characteristic tensile vibration peaks of O-H and N-H [28,29]. The above results indicated that PDA was successfully incorporated into the hydrogel through solution soaking.

In order to study the effect of DA concentration in solution on the structure of hydrogel, SEM was used to investigate the internal structural changes of the PDA-CA-PAM hydrogels after immersion in the solution with different DA concentrations. Figure 1b–e showed SEM images of the cross sections of CA-PAM and PDA-CA-PAM hydrogels. Comparing the SEM images of the hydrogels, it can be seen intuitively that the CA-PAM and PDA-CA-PAM hydrogels all had a good three-dimensional porous network structure. With the increase in DA concentration, the pore density of the gel increased, and the pore size gradually decreased. Presumably, it is because PDA entered the interior of the gel network during the immersion and formed a new internal structure through non-covalent bonding, further dividing the original internal pores. The structural variations may potentially affect the properties of the hydrogel.

### 3.2. Mechanical Properties and Rheological Analysis

**Figure 2:**
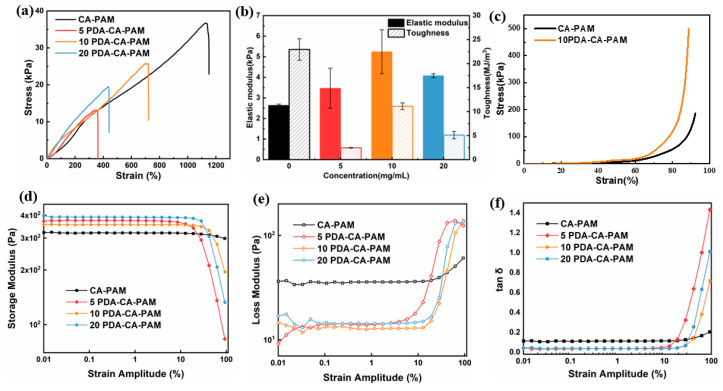
(**a**) Tensile stress–strain curve. (**b**) Elastic modulus and toughness of CA-PAM and PDA-CA-PAM hydrogels. (**c**) The compressive stress–strain curve of the CA-PAM and 10 PDA-CA-PAM hydrogels. (**d**) Storage modulus, (**e**) loss modulus, and (**f**) loss angle of the hydrogels.

Excellent mechanical properties are important for anti-adhesion hydrogels, which enable them to act as support and a barrier in the uterine cavity. Tensile and compression tests and rheological analyses were carried out on the hydrogels, as shown in Figure 2. Figure 2a shows that compared with CA-PAM, the elongation rate and tensile strength of PDA-CA-PAM decreased, which was because the doped PDA limited the movement and extension of the polymer chain during stretching. The elongation at break of PDA-CA-PAM increased with the DA concentration. The reason was that during the PDA coating process, the polymerized PDA chain infiltrated the hydrogel network, increasing the number of non-covalent bonding sites within the gel, and when the molecular chain slipped during stretching, new non-covalent bonds were formed. Consequently, this resulted in an increase in elongation at break. When the concentration of DA exceeded 10 mg/mL, the elongation started to decrease. It was speculated that this decrease was due to the uneven cross-linking within the hydrogel caused by excessive PDA doping. The excess intermolecular hydrogen bonds generated defects in localized areas, making the hydrogel more prone to cracking during stretching, leading to a decline in its mechanical properties [30,31].

Figure 2b shows the corresponding elastic modulus and toughness calculated from the tensile stress–strain curve. It can be seen that the elastic modulus of the original CA-PAM hydrogel was slightly increased after coating with PDA, which was because the internal structure of the PDA-CA-PAM hydrogel became closer due to the PDA coating. When the concentration of DA was 10 mg/mL, the elongation at break of 10 PDA-CA-PAM was 720%, which indicated excellent flexibility. The elastic modulus (5.24 ± 1.06 kPa) of the 10 PDA-CA-PAM hydrogel was close to that of natural rat uterine tissue [32], proving the potential to be used in uterine cavity anti-adhesion.

The compressive stress–strain curve of the 10 PDA-CA-PAM hydrogel is shown in Figure 2c. It can be found that the compression strain of the 10 PDA-CA-PAM hydrogel was 89% when the compression stress was as high as 0.5 MPa. After PDA doping, the hydrogel had better compression performance because of the denser internal network structure, indicating that it could smoothly play a supporting role on the uterine cavity wound.

To further investigate the effect of PDA doping on the internal structure of the hydrogels, the shear viscoelasticity was analyzed at 37 °C. Figure 2d,e shows the storage modulus G′ and loss modulus G″ of the hydrogels as a function of the shear strain amplitude. Within the strain amplitude range of 0.01~10%, because of the denser structure due to PDA doping, PDA-CA-PAM hydrogels had higher G′ and lower G″ compared to CA-PAM, showing better elasticity. When the strain amplitude was greater than 10%, the internal network structure of the hydrogels lost stability. Among these, the intersection points of G′ and G″ of the 10 PDA-CA-PAM hydrogel was more posterior, indicating that it had the highest stability. Overall comparison, it can also be found that the 10 PDA-CA-PAM hydrogel had lower G″, with a more stable internal structure, which can be confirmed by the results of tensile test.

Figure 2f shows the loss factor of hydrogels as a function of the shear strain amplitude. When the strain amplitude was less than 10%, the tan δ of the PDA-CA-PAM hydrogels was lower than that of CA-PAM, indicating better elasticity. When the strain amplitude was greater than 10%, the tan δ rose rapidly. Among them, the 10 PDA-CA-PAM hydrogel had the lowest tan δ, and its rapidly rising initial strain was more posterior, proving that it had relatively stronger elasticity. Based on the above analysis, among several PDA-doped hydrogels, the 10 PDA-CA-PAM hydrogel exhibited the lowest loss modulus and loss angle, indicating that its internal network possessed relatively better elasticity and shape stability.

### 3.3. Adhesive Performance

**Figure 3:**
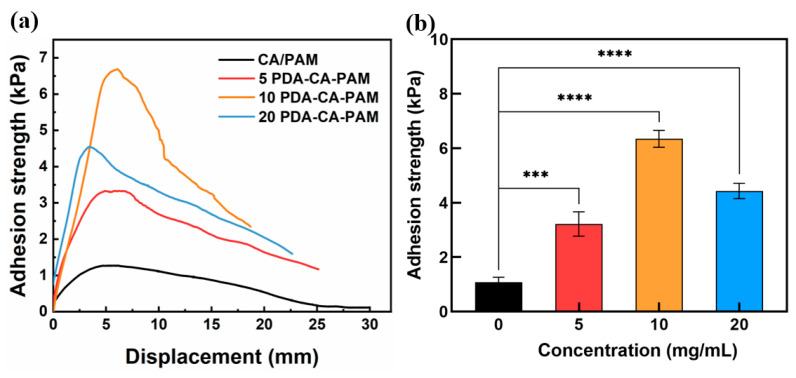
(**a**) Adhesion stress–displacement curves and (**b**) maximum adhesion strength of CA-PAM and PDA-CA-PAM hydrogels applied to porcine skin. (*p* < 0.001 was marked as “***” and *p* < 0.0001 was marked as “****”).

In order to obtain the adhesive properties of the hydrogel, a lap shear test was performed. The anti-adhesive hydrogel required good tissue adhesion to ensure stable attachment to the wound site. The adhesion stress–displacement curves are shown in Figure 3a, and the maximum value on the adhesion curve was extracted to obtain Figure 3b. It can be seen that the adhesion strength increased with the increase in DA concentration. When the concentration of DA reached 10 mg/mL, the adhesion strength between the PDA-CA-PAM hydrogel and pig skin reached 6.35 ± 0.31 kPa, which was higher than the adhesion strength of commercial tissue adhesive fibrin glue (about 5 kPa) [33]. The 10 PDA-CA-PAM hydrogel also showed good adhesion to other materials (Figure S1).

The good adhesion performance of the PDA-CA-PAM hydrogel was mainly attributed to the catechol, amino, and carboxyl groups of PDA, which could generate hydrogen bonds with biological tissue interfaces and mimic the adhesion function of mussel. The 10 PDA-CA-PAM hydrogel had the best adhesion properties. However, when the concentration of DA exceeded 10 mg/mL, the adhesive strength began to decline. We speculated that this was due to excessive PDA chains competing with acrylamide (AM) for the free radicals generated by ammonium persulfate (APS), resulting in the excessive polymerization of PDA. Consequently, the number of phenolic hydroxyl groups available for tissue adhesion decreased, ultimately leading to a deterioration in the adhesive performance [34,35]. Because of the presence of microvillous structures and adhesive proteins on the uterine mucosa, we speculate that these will contribute to the formation of a stronger adhesion between the hydrogel and the mucosal surface. Due to the 10 PDA-CA-PAM hydrogel having the highest elastic modulus (5.24 ± 1.06 kPa), as well as the best adhesion ability (6.35 ± 0.31 kPa), it was selected for further investigation.

### 3.4. Swelling and Biodegradation

**Figure 4:**
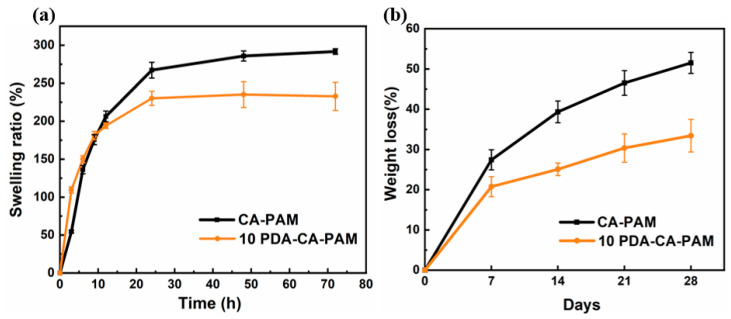
(**a**) Swelling rate and (**b**) degradation rate of the CA-PAM and 10 PDA-CA-PAM hydrogels.

A hydrogel used for preventing adhesion in uterine cavity wounds requires a good swelling property to effectively absorb wound exudate and ensure wound cleanliness. The swelling properties of both hydrogels were tested using PBS as the simulated body fluid. The 10 PDA-CA-PAM hydrogel also showed a certain swelling ability, providing a guarantee for the absorption of secretions around the uterine cavity wound. As shown in Figure 4a, after 72 h of swelling in PBS solution, the maximum swelling rate of 10 PDA-CA-PAM reached 235% and kept the shape intact (Figure S2). Compared to that of CA-PAM (292%), the swelling ability of 10 PDA-CA-PAM decreased because PDA doping resulted in more entanglement of molecular chains, lower extension ability of molecular chain segments, and less space for storing water molecules. Even so, the swelling rate could still reach more than 200%, and it had the ability to absorb wound exudate.

As a kind of body implant material, the degradation rate of anti-adhesion hydrogels should, ideally, match the tissue regeneration rate. Since alginate was degraded via Na^+^ and Ca^2+^ ion exchange, and PAM was degraded via the reaction between the disulfide bond and cysteine [36,37], cysteine was added into PBS at 37 °C to further simulate body fluid for a more realistic degradation rate. Figure 4b shows that the 10 PDA-CA-PAM hydrogel had a more stable degradation rate, with a loss of about 33.5% after 4 weeks. The incorporation of PDA could increase the cross-linking density of the 10 PDA-CA-PAM hydrogel network, reduce the inside reactant content, and form a coating to reduce the contact area between the gel matrix and the simulated body fluid so as to achieve more stable degradation.

Considering the comprehensive swelling and degradation curve, it can be observed that the swelling rate of the 10 PDA-CA-PAM hydrogel decreased partially by the third day. At the same time, a certain level of degradation had already occurred, resulting in a decrease in the matrix content and thus less moisture absorption, which manifested as a reduction in the swelling rate. In contrast, the CA-PAM hydrogel did not exhibit this phenomenon. It is speculated that larger internal pores allowed for more water absorption, leading to a slower approach to the swelling limit and a smaller impact of degradation on mass change.

### 3.5. Hydrophilicity and Hemostasis Performance

**Figure 5:**
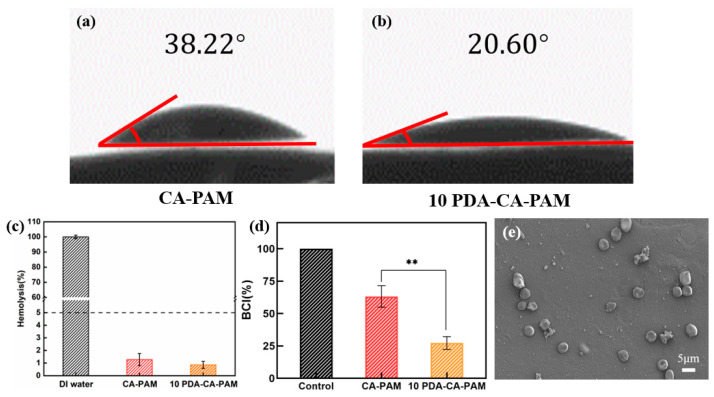
(**a**,**b**) Hydrophilic angles, (**c**) hemolysis rate, and (**d**) BCI index of the CA-PAM and 10 PDA-CA-PAM hydrogels. (**e**) SEM image of blood cells attached to the surface of the 10 PDA-CA-PAM hydrogel. (*p* < 0.01 was marked as “**”).

Anti-adhesion hydrogels require good hydrophilicity in order to adhere to cells, and it is necessary to test the water contact angle. As shown in Figure 5a,b, the 10 PDA-CA-PAM hydrogel had a water contact angle of 20.60°, while the CA-PAM hydrogel had a water contact angle of 38.22°. Due to the large number of hydrophilic groups present in the PDA chain segment, its presence reduced the water contact angle and enhanced hydrophilicity.

According to the international standard (ISO/TR 7405), the hemolysis rate of anti-adhesion hydrogel in contact with blood should be less than 5.0% before it can be used normally. It was necessary to perform in vitro hemolysis assays to assess the hemolytic effect of the 10 PDA-CA-PAM hydrogel. As shown in Figure 5c, the hemolysis rates of the CA-PAM and 10 PDA-CA-PAM hydrogels were both lower than the black dashed line of 5%, and the latter was lower, indicating that the doping PDA could afford the hydrogel better blood compatibility. The results showed that the 10 PDA-CA-PAM hydrogel had good blood compatibility.

Hemostasis is the first step in the healing process of the uterine cavity wound to prevent excessive blood loss before regrowth of the cell and tissue [38], and an ideal anti-adhesion hydrogel requires good hemostasis ability. The hemostatic ability of 10 PDA-CA-PAM was evaluated via the blood clotting index (BCI). Figure 5d shows that, as well as excellent mechanical and adhesion properties, the 10 PDA-CA-PAM hydrogel also has hemostatic ability. Compared with the control group, the coagulation index of 10 PDA-CA-PAM was 27.27 ± 4.91, and that of CA-PAM was 63.02 ± 8.36. The results also showed that the 10 PDA-CA-PAM hydrogel had a lower BCI index than the commercially available gauze (40–55, BCI) [39]. The PDA coating on the surface of the 10 PDA-CA-PAM hydrogel can improve the hydrogel’s hydrophilicity, promote more blood cells to adhere to the surface, and has better hemostatic ability.

The 10 PDA-CA-PAM hydrogel may have a potential effect on the morphological changes of blood cells, which was investigated via SEM. Figure 5e shows that blood cells have a certain degree of aggregation and adhesion on the surface of the 10 PDA-CA-PAM hydrogel but no abnormal deformation or aggregation, which further proves that the hydrogel has good blood compatibility and hemostatic ability.

### 3.6. Cell Proliferation, Viability, and Attachment

**Figure 6:**
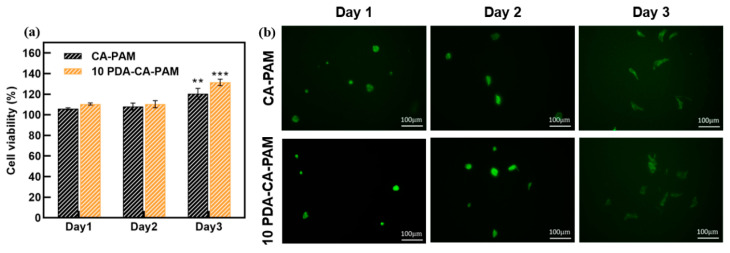
(**a**) Cell activity of fibroblasts cultured on the surface of the CA-PAM and 10 PDA-CA-PAM hydrogels on day 1, 2, and 3. (**b**) Fluorescently stained images of fibroblasts attached to the surface of the hydrogels on day 1, day 2, and day 3. *p* < 0.01 was marked as “**” and *p* < 0.001 was marked as “***”.

Good biocompatibility is the basic characteristic of anti-adhesion hydrogels. The cytotoxicity of the extract of the CA-PAM and 10 PDA-CA-PAM hydrogel was evaluated via CCK-8 assay using NIH-3T3 cells. Figure 6a shows that NIH-3T3 cells were cultured on the surface of the two hydrogels for 1 d, 2 d, and 3 d. Compared to the control group, cells co-cultured with hydrogel exhibited enhanced cell viability, indicating that the cells could grow and proliferate normally on the surface, and the hydrogels had good cytocompatibility. Compared with CA-PAM, the cell viability of the 10 PDA-CA-PAM surface was higher because the PDA coating afforded the surface better hydrophilicity and promoted cell adhesion and proliferation. This indicated that the 10 PDA-CA-PAM hydrogel exhibited good cytocompatibility.

Fibroblasts play a very important role in the formation of postoperative adhesion, and the anti-adhesion hydrogel should not induce excessive fibroblast adhesion and uncontrolled proliferation in the initial stage [40]. It is necessary to observe the cells’ adhesion on the surface of the hydrogel via fluorescence staining. As shown in Figure 6b, NIH-3T3 cells could gradually spread on the surface of the CA-PAM and 10 PDA-CA-PAM hydrogels without abnormal proliferation or agglomeration. Due to the better surface hydrophilicity caused by the PDA coating, more NIH-3T3 cells were attached to the surface of the 10 PDA-CA-PAM hydrogel, but they were gradually dispersed over culture time without excessive attachment. The good biocompatibility further indicated that 10 PDA-CA-PAM would be a kind of anti-adhesion material with application potential.

## 4. Conclusions

In summary, a multifunctional hydrogel with good mechanical properties, adhesion, and hemostasis has been prepared. The hydrogel was prepared on the basis of the dual-network CA-PAM hydrogel via DA solution immersion. It has been shown that PDA can regulate the hydrogel structure through non-covalent hydrogen bonding, thereby modulating the mechanical properties. Hydrogels with desirable mechanical properties can be obtained at specific DA solution concentrations. The presence of PDA coating optimizes the degradation rate, blood compatibility, and cytocompatibility of the hydrogel to meet the requirements of implantation. Furthermore, the desirable performance demonstrated by the hydrogel in in vitro testing can provide a solid foundation for its further application. Overall, this hydrogel contains almost all the desired beneficial properties, and has great clinical application potential in the prevention of postoperative re-adhesion.

## Data Availability

The raw/processed data required to reproduce these findings cannot be shared at this time as these data also form part of an ongoing study.

## Supplementary Materials

The following supporting information can be downloaded at https://www.mdpi.com/article/10.3390/polym15234498/s1. Figure S1: Adhesion of the 10 PDA-CA-PAM hydrogel to other materials; Figure S2: Comparison between the CA-PAM and PDA-CA-PAM hydrogels before and after swelling.

## References

[1] Yu D., Wong Y.M., Cheong Y., Xia E.L., Li T.C. (2008). Asherman syndrome-one century later. Fertil. Steril..

[2] Sardo A.D., Calagna G., Scognamiglio M., O’Donovan P., Campo R., De Wilde R.L. (2016). Prevention of intrauterine post-surgical adhesions in hysteroscopy. A systematic review. Eur. J. Obstet. Gynecol. Reprod. Biol..

[3] Wei C., Pan Y.B., Zhang Y.L., Dai Y.D., Jiang L.L., Shi L.B., Yang W.J., Xu S.Q., Zhang Y.Y., Xu W.Z. (2020). Overactivated sonic hedgehog signaling aggravates intrauterine adhesion via inhibiting autophagy in endometrial stromal cells. Cell Death Dis..

[4] Deans R., Abbott J. (2010). Review of Intrauterine Adhesions. J. Minim. Invasive Gynecol..

[5] Salma U., Xue M., Sayed A.S.M., Xu D.B. (2014). Efficacy of Intrauterine Device in the Treatment of Intrauterine Adhesions. Biomed. Res. Int..

[6] Lin X.N., Zhou F., Wei M.L., Yang Y., Li Y., Li T.C., Zhang S.Y. (2015). Randomized, controlled trial comparing the efficacy of intrauterine balloon and intrauterine contraceptive device in the prevention of adhesion reformation after hysteroscopic adhesiolysis. Fertil. Steril..

[7] Zhang E.S., Yang J.H., Wang K., Song B.Y., Zhu H., Han X.F., Shi Y.J., Yang C.B., Zeng Z.P., Cao Z.Q. (2021). Biodegradable Zwitterionic Cream Gel for Effective Prevention of Postoperative Adhesion. Adv. Funct. Mater..

[8] Tonguc E.A., Var T., Yilmaz N., Batioglu S. (2010). Intrauterine device or estrogen treatment after hysteroscopic uterine septum resection. Int. J. Gynecol. Obstet..

[9] Erdi M., Saruwatari M.S., Rozyyev S., Acha C., Ayyub O.B., Sandler A.D., Kofinas P. (2023). Controlled Release of a Therapeutic Peptide in Sprayable Surgical Sealant for Prevention of Postoperative Abdominal Adhesions. ACS Appl. Mater. Interfaces.

[10] Zou Y.K., Yue P.P., Cao H.K., Wu L.Q., Xu L., Liu Z.Z., Wu S.Q., Ye Q.F. (2023). Biocompatible and biodegradable chitin-based hydrogels crosslinked by BDDE with excellent mechanical properties for effective prevention of postoperative peritoneal adhesion. Carbohyd. Polym..

[11] Orhue A.A.E., Aziken M.E., Igbefoh J.O. (2003). A comparison of two adjunctive treatments for intrauterine adhesions following lysis. Int. J. Gynecol. Obs..

[12] Gibbs D.M.R., Black C.R.M., Dawson J.I., Oreffo R.O.C. (2016). A review of hydrogel use in fracture healing and bone regeneration. J. Tissue Eng. Regen. Med..

[13] Zhang Y.S., Khademhosseini A. (2017). Advances in engineering hydrogels. Science.

[14] Naahidi S., Jafari M., Logan M., Wang Y.J., Yuan Y.F., Bae H., Dixon B., Chen P. (2017). Biocompatibility of hydrogel-based scaffolds for tissue engineering applications. Biotechnol. Adv..

[15] Burdick J.A., Murphy W.L. (2012). Moving from static to dynamic complexity in hydrogel design. Nat. Commun..

[16] Seliktar D. (2012). Designing Cell-Compatible Hydrogels for Biomedical Applications. Science.

[17] Fei Z., Xin X., Fei H., Cui Y.C. (2020). Meta-analysis of the use of hyaluronic acid gel to prevent intrauterine adhesions after miscarriage. Eur. J. Obstet. Gynecol. Reprod. Biol..

[18] Kim T., Ahn K.H., Choi D.S., Hwang K.J., Lee B.I., Jung M.H., Kim J.W., Kim J.H., Cha S.H., Lee K.H. (2012). A Randomized, Multi-Center, Clinical Trial to Assess the Efficacy and Safety of Alginate Carboxymethylcellulose Hyaluronic Acid Compared to Carboxymethylcellulose Hyaluronic Acid to Prevent Postoperative Intrauterine Adhesion. J. Minim. Invasive Gynecol..

[19] Park D.Y., Chung H.J., Sim N.S., Jo K.H., Kim D.H., Kim C.H., Yoon J.H. (2016). Comparison of calcium alginate and carboxymethyl cellulose for nasal packing after endoscopic sinus surgery: A prospective, randomised, controlled single-blinded trial. Clin. Otolaryngol..

[20] Carruthers A., Carruthers J. (2007). Non-animal-based hyaluronic acid fillers: Scientific and technical considerations. Plast. Reconstr. Surg..

[21] Sun J.Y., Zhao X.H., Illeperuma W.R.K., Chaudhuri O., Oh K.H., Mooney D.J., Vlassak J.J., Suo Z.G. (2012). Highly stretchable and tough hydrogels. Nature.

[22] Wang J., Chen Y.P., Zhou G.S., Chen Y.Y., Mao C.B., Yang M.Y. (2019). Polydopamine-Coated Antheraea pernyi (A. pernyi) Silk Fibroin Films Promote Cell Adhesion and Wound Healing in Skin Tissue Repair. ACS Appl. Mater. Interfaces.

[23] Jing X., Mi H.Y., Lin Y.J., Enriquez E., Peng X.F., Turng L.S. (2018). Highly Stretchable and Biocompatible Strain Sensors Based on Mussel-Inspired Super-Adhesive Self-Healing Hydrogels for Human Motion Monitoring. ACS Appl. Mater. Interfaces.

[24] Sun P.Y., Wang J., Yao X., Peng Y., Tu X.X., Du P.F., Zheng Z., Wang X.L. (2014). Facile Preparation of Mussel-Inspired Polyurethane Hydrogel and Its Rapid Curing Behavior. ACS Appl. Mater. Interfaces.

[25] Lee H., Dellatore S.M., Miller W.M., Messersmith P.B. (2007). Mussel-inspired surface chemistry for multifunctional coatings. Science.

[26] Sileika T.S., Kim H.D., Maniak P., Messersmith P.B. (2011). Antibacterial Performance of Polydopamine-Modified Polymer Surfaces Containing Passive and Active Components. ACS Appl. Mater. Interfaces.

[27] Ding Y.H., Floren M., Tan W. (2016). Mussel-inspired polydopamine for bio-surface functionalization. Biosurf. Biotribol..

[28] Suneetha M., Rao K.M., Han S.S. (2019). Mussel-Inspired Cell/Tissue-Adhesive, Hemostatic Hydrogels for Tissue Engineering Applications. Acs Omega.

[29] Xie Z.H., Li H., Mi H.Y., Feng P.Y., Liu Y.J., Jing X. (2021). Freezing-tolerant, widely detectable and ultra-sensitive composite organohydrogel for multiple sensing applications. J. Mater. Chem. C.

[30] Fan L., Xie L., Zheng Y.P., Wei D.X., Yao D.D., Zhang J., Zhang T.D. (2020). Antibacterial, Self-Adhesive, Recyclable, and Tough Conductive Composite Hydrogels for Ultrasensitive Strain Sensing. ACS Appl. Mater. Interfaces.

[31] Yan M., Shi J.F., Tang S., Zhou G.H., Zeng J.X., Zhang Y.X., Zhang H., Yu Y., Guo J. (2021). Preparation of high-strength and high-toughness biomass medical films based on a polydopamine dynamically united calcium alginate/carboxymethyl chitosan dual network. New J. Chem..

[32] Xiao B., Yang W.J., Lei D., Huang J.F., Yin Y.P., Zhu Y.Q., You Z.W., Wang F., Sun S.H. (2019). PGS Scaffolds Promote the In Vivo Survival and Directional Differentiation of Bone Marrow Mesenchymal Stem Cells Restoring the Morphology and Function of Wounded Rat Uterus. Adv. Healthc. Mater..

[33] Lih E., Lee J.S., Park K.M., Park K.D. (2012). Rapidly curable chitosan-PEG hydrogels as tissue adhesives for hemostasis and wound healing. Acta Biomater..

[34] Liang M., He C.P., Dai J.D., Ren P.F., Fu Y.F., Wang F.M., Ge X., Zhang T.Z., Lu Z.H. (2020). A high-strength double network polydopamine nanocomposite hydrogel for adhesion under seawater. J. Mater. Chem. B.

[35] Bu Y.Z., Pandit A. (2022). Cohesion mechanisms for bioadhesives. Bioact. Mater..

[36] Liu J.Y., Pang Y., Zhang S.Y., Cleveland C., Yin X.L., Booth L., Lin J.Q., Lee Y.A.L., Mazdiyasni H., Saxton S. (2017). Triggerable tough hydrogels for gastric resident dosage forms. Nat. Commun..

[37] Li J.Y., Suo Z.G., Vlassak J.J. (2014). Stiff, strong, and tough hydrogels with good chemical stability. J. Mater. Chem. B.

[38] Qu J., Zhao X., Liang Y.P., Zhang T.L., Ma P.X., Guo B.L. (2018). Antibacterial adhesive injectable hydrogels with rapid self-healing, extensibility and compressibility as wound dressing for joints skin wound healing. Biomaterials.

[39] Ahmadian Z., Correia A., Hasany M., Figueiredo P., Dobakhti F., Eskandari M.R., Hosseini S.H., Abiri R., Khorshid S., Hirvonen J. (2021). A Hydrogen-Bonded Extracellular Matrix-Mimicking Bactericidal Hydrogel with Radical Scavenging and Hemostatic Function for pH-Responsive Wound Healing Acceleration. Adv. Healthc. Mater..

[40] Liu X.M., Wang H.H., She J.J., Zhang Q., Hu Q.Y., Li D.D., Wu H.L., Ye X.F., Diao R.Y., Shi X.T. (2023). An anti-fibroblast adhesion and anti-inflammatory hydrogel film combined with VEGF for intrauterine adhesion prevention. Chem. Eng. J..

