# Supplementary material for: Mussel-Inspired Calcium Alginate/Polyacrylamide Dual Network Hydrogel: A Physical Barrier to Prevent Postoperative Re-Adhesion
Source: Polymers (Basel). 2023 Nov 23;15(23):4498. doi: 10.3390/polym15234498 (PMC10708265; doi:10.3390/polym15234498)
Supplement: Supplementary file 1 [file polymers-15-04498-s001.zip › polymers-2675426-supplementary.pdf]

Mussel-inspired calcium alginate/polyacrylamide dual network hydrogel: a physical barrier to prevent postoperative re-adhesion

Zekun Su[1], Beibei Xue[1], Chang Xu[2], Yue Kang[3], Xufeng Dong[1*]

[1] School of Materials Science and Engineering, Dalian University of Technology, Dalian 116024, China.

[2] Institute of Cardio-Cerebrovascular Medicine, Central Hospital of Dalian University of Technology, Dalian 116089, China.

[3] Department of Breast Surgery, Liaoning Cancer Hospital & Institute, Shenyang 110042, China.

* Corresponding author.

E-mail addresses: dongxf@dlut.edu.cn (X. Dong)

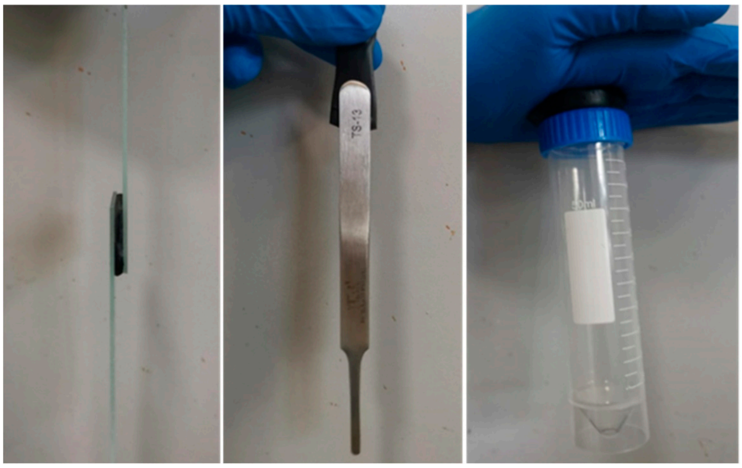

Figure S1. Adhesion of 10 PDA-CA-PAM hydrogel to other materials.

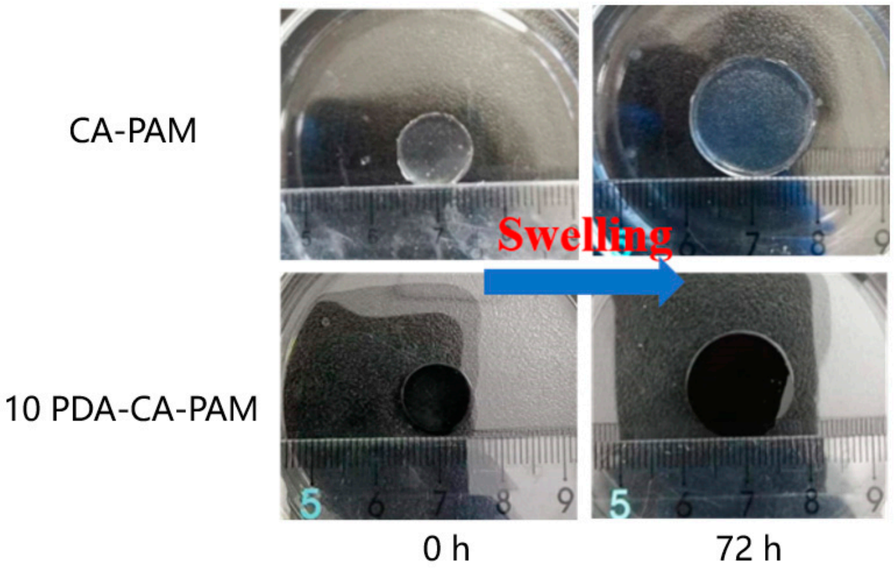

Figure S2. Comparison between CA-PAM and PDA-CA-PAM hydrogel before and after swelling.
